# Membrane nanotubes are ancient machinery for cell-to-cell communication and transport. Their interference with the immune system

**DOI:** 10.1007/s42977-020-00062-0

**Published:** 2021-02-08

**Authors:** János Matkó, Eszter Angéla Tóth

**Affiliations:** 1 Department of Immunology, Institute of Biology, Eötvös Loránd University, H-1117 Pázmány Péter sétány 1/C, Budapest, Hungary; 2 ATRC Aurigon Toxicological Research Center, H-2120 Pálya utca 2, Dunakeszi, Hungary

**Keywords:** Membrane nanotubes, Intercellular transport, Long-distance signaling, (Archae) Bacteria, Immunomodulation, Nanoparticle therapy

## Abstract

Nanotubular connections between mammalian cell types came into the focus only two decades ago, when “live cell super-resolution imaging” was introduced. Observations of these long-time overlooked structures led to understanding mechanisms of their growth/withdrawal and exploring some key genetic and signaling factors behind their formation. Unbelievable level of multiple supportive collaboration between tumor cells undergoing cytotoxic chemotherapy, cross-feeding” between independent bacterial strains or “cross-dressing” collaboration of immune cells promoting cellular immune response, all via nanotubes, have been explored recently. Key factors and "calling signals" determining the spatial directionality of their growth and their overall in vivo significance, however, still remained debated. Interestingly, prokaryotes, including even ancient *archaebacteria*, also seem to use such NT connections for intercellular communication. Herein, we will give a brief overview of current knowledge of membrane nanotubes and depict a simple model about their possible “historical role”.

## Background

**Fig. 1:**
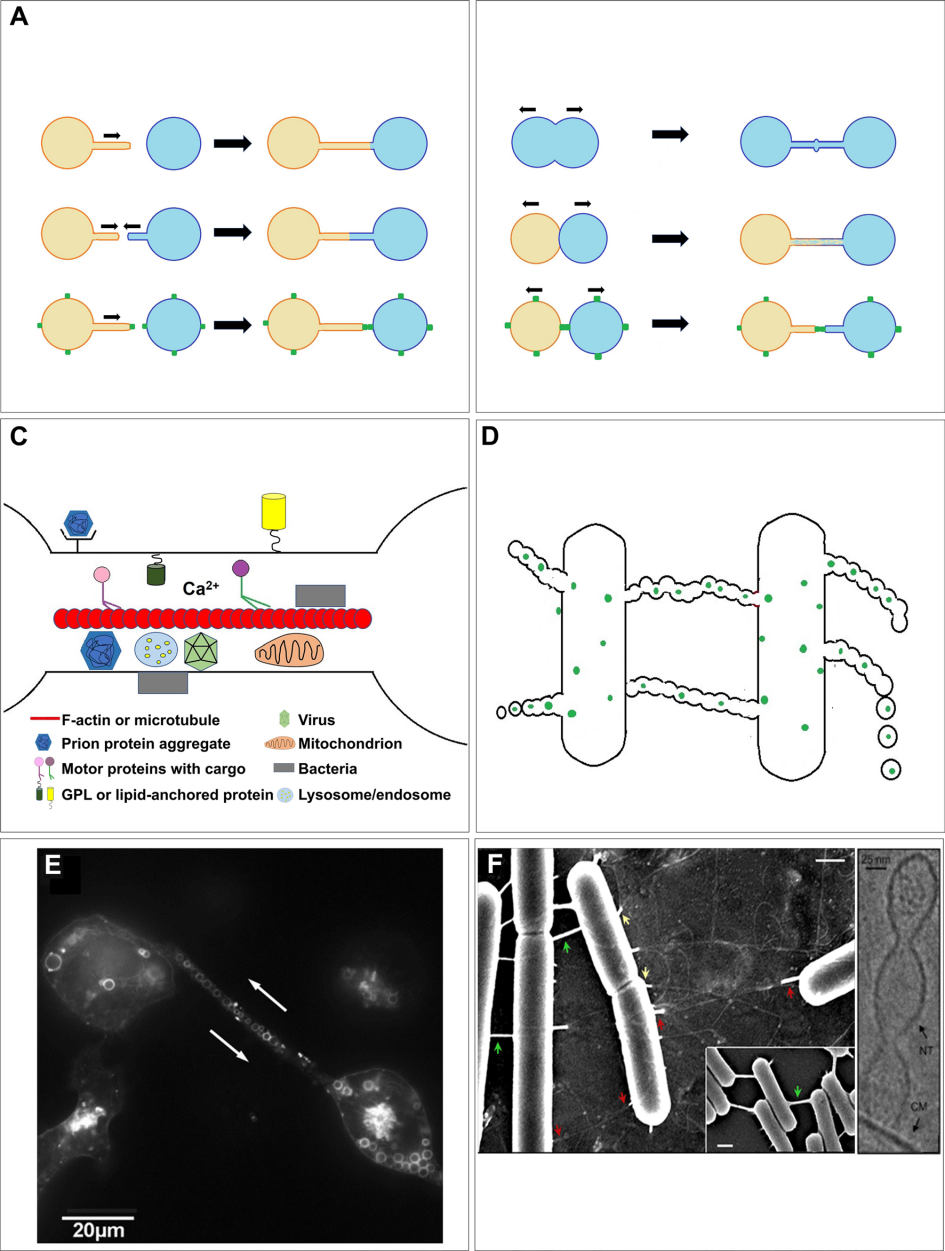
How the pro- and eukaryotic nanotubes are created and look like? The NTs can form as protrusions from a cell upon an initiating signal (*A, upper panel*). Both cells can grow NTs to each other (*A, middle panel*). One cell can grow nanotube toward another one which connects the cells through connexin dimerization (*A, lower panel*). NTs may also form upon cell division accompanied by a marker called "division ring” (*B, upper panel*). Cells may also form nanotubules after a close physical contact, such as immunological synapse (IS) (*B, middle panel*) or connexin-mediated junctions (*B, lower panel*). The so-called tunneling nanotubes (TNTs) may be platforms for intercellular transfer of viruses, bacteria, prions, tau proteins, membrane protein patches, vesicles, ions, miRNA, etc. (*panel C*). Bacteria also form nanotubes allowing intercellular transport with a special morphological feature of series of fused microvesicles (*panel D*). High-resolution in vitro images of eukaryotic (*B cell*) (*panel E*) and bacterial (*Bacillus Subtilis*) (*panel F*) nanotubes are also shown. ( Copyright permissions from Osteikoetxea-Molnar et al. Cell Mol Life Sci 2016; Dubey et al. Dev Cell 2016)

Membrane nanotubule structures, named also as “cytonemes” (Ramírez-Weber and Kornberg 1999), “membrane tethers”, “nanotubes (NTs)”, “tunneling nanotubes (TNTs)” (Iglič et al. 2003; Onfelt et al. 2006; Rustom et al. 2004) or “tumor microtubes” (TMs) (Lou et al. 2012b; Osswald et al. 2015) are thin cellular protrusions connecting two (or rarely more) neighboring cells from short to long distance (10–150 µm). Nanotubes were first defined by (Ramírez-Weber and Kornberg 1999) studying the mechanisms involved in the development of drosophila wing and eye imaginal disks. Later, utilizing the newly introduced “live cell super-resolution imaging modalities” allowed observations at as low as 30–50 nm spatial resolution level, at closely physiological conditions. Pioneering works explored various types of nanotubes on different eukaryotic cell types, such as red blood cells, neurons, immune cells and various kinds of tumor cells (Davis and Sowinski 2008; Iglič et al. 2003; Kralj-Iglič et al. 2001; Lou et al. 2012b; Osswald et al. 2015; Rustom et al. 2004). It turned out that these NTs, besides conducting molecular or electric signals (Abounit and Zurzolo 2012; Rainy et al. 2013; Smith et al. 2011; Watkins and Salter 2005) are also enabled to form active channels (TNTs) between the conne*c*ted cells (for distance as long as 100 µm) across which organelles, microvesicles, proteins, nucleic acids, micro-RNA, prions, viruses or lipid droplets can be transported, often including microtubule-based, motor protein driven transfer processes(Gousset et al. 2009; Gurke et al. 2008; Halász et al. 2018; Osteikoetxea-Molnár et al. 2016). Furthermore, these NTs could be organized into an *extended network* of tens or more NTs, especially in the case of neural–neural, tumor–tumor, or tumor–stem cell connections (Ariazi et al. 2017; Garden and La Spada 2012; Thayanithy et al. 2014). Although such extended networks are rare between immune cells, but instead a very intensive molecular and vesicular transport could be observed via nanotubes between them (Halász et al. 2018; Osteikoetxea-Molnár et al. 2016; Rainy et al. 2013; Sowinski et al. 2008). Such processes sometimes bridge the extremely long distance, as demonstrated by in vivo imaging of intercellular MHC transfer in inflamed mouse cornea (Chinnery et al. 2008; Seyed-Razavi et al. 2013). These observations all together led to some general concepts about NTs. It was proposed first (Kornberg 2019; Zhang and Scholpp 2019) that “*cells in trouble or in development” may grow NTs toward other cells transmitting “calling signals” for them*. This concept has recently been supported by many experimental data for neuronal and tumor cell networks or tumor–stem cell networks, alike. Starting from this working hypothesis, by now we learned a lot mostly about eukaryotic NTs, and less about bacterial NT networks (Fig. 1), but still many questions waiting for answers.

Next, first we summarize the major lessons what we learned from the two decades of nanotube research about the mechanisms of their formation and the factors controlling it. Then, we briefly analyze the advantages of this knowledge in biomedicine, focusing on immunobiology. We will also discuss the possibilities of such intercellular communication in the ancient eras, briefly overviewing bacterial communication pathways, and intend to depict a simple model about the possible historical role of nanotubes.

## What we have learned from the early eukaryote nanotube studies?

First, we learned that such nanotubular structures can dynamically grow out from the cells or withdrawn depending on their actual physiological status controlled by signals received from the environment and regulating actin polymerization/redistribution (Davis and Sowinski 2008; Delage et al. 2016; Drab et al. 2019; Hanna et al. 2017; Kimura et al. 2012; Onfelt et al. 2006; Osteikoetxea-Molnár et al. 2016; Rustom et al. 2004). These basic studies confirmed that the major molecular skeleton of the NTs is F-actin, the polymerization of which is the driving force of NT growth in most cases. However, some of the NTs, the so-called “at both ends open-ended, tunneling” nanotubes (TNTs) may also contain a significant level of microtubules inside(Onfelt et al. 2006; Osteikoetxea-Molnár et al. 2016; Veranic et al. 2008). That will allow transport of a wide variety of cargos through actin- or microtubule-based motor protein driven transport.

Second, further biochemical and biophysical studies have shown that there is a molecular level control as well on NT-formation: the actual lipid composition of the plasma membrane, the ratio of raftophilic/raftophobic lipids (Delage and Zurzolo 2013; Lokar et al. 2012; Tóth et al. 2017), membrane-associated BAR-domain proteins controlling membrane curvature(McMahon and Gallop 2005; Zhao et al. 2011), the interaction of membrane integrin protein chains with their extracellular matrix counterparts (Osteikoetxea-Molnár et al. 2016) all are important determinants of nanotube growth and mechanical properties of cell membranes. A direct contribution of the membrane-bound insulin receptor substrate protein 53 *(IRSp53)* N-terminal domain to bending of the plasma membrane and clustering of PI(4,5)P2 nicely demonstrated that these molecular interactions are among the key mechanisms controlling the formation of protrusions and tunneling nanotubes (Saarikangas et al. 2009).

Third, we learned that the tunneling version of nanotubes (TNTs) may behave as long channels bridging two cells and allowing transport of ions, molecules, prions, viruses, micro-RNA and most interestingly even intact organelles (e.g., mitochondria, lysosome) or various intracellular membranous microvesicles (iMVs) (see Fig. 1) (Lou et al. 2012a; Osswald et al. 2015; Osteikoetxea-Molnár et al. 2016)*.* Intercellular transport of mitochondria via TNTs was interpreted in an interesting model (Scholkmann 2016) as a "long-range signaling mechanism” where the two connected cells can exchange energy and signals through the connective network of mitochondria, confirmed by experimental data, as well. The intercellular exchange of mitochondria and other molecular factors gained a special medical interest in tumor biomedicine (Desir et al. 2018; Lou et al. 2012a, 2018; Osswald et al. 2015; Pasquier et al. 2013) and also in cell death research (Arkwright et al. 2010; Beum et al. 2008; Wang and Gerdes 2015), because it was demonstrated that the intercellularly transported mitochondria could prevent recipient cells from cell death. Recently a surprising study reported on so-called “*mitochondrial nanotunnels*”, which are double membrane-covered (containing both inner and outer membranes of mitochondria) cellular protrusions observed between human, rat and monkey cardiomyocytes, skeletal muscle and kidney cell types (Vincent et al. 2017). This observation raises already the possibility that predecessors of the current nanotubes in multicellular organisms could have been existed ages earlier.

Finally, we also learned how can we envision and monitor these nanotubular structures and their function in cell cultures or in vivo, but a fundamental doubt still remained: regarding their relatively low abundance (15–40% of cells are NT +) in in vitro cell cultures, we can ask whether their function might have a significant impact on mammalian cell functions in vivo, at the system level, in health and disease. In addition, there is another coupled question: how far the two cells should be located spatially (in vivo) to be eligible to participate in such nanotube networks? This question became especially interesting, since recently, to our surprise, thin and relatively short nanotubular structures have been detected in ex vivo mouse brain tumor metastases at high density, utilizing the technical advantages of lattice light-sheet microscopy (Parker et al. 2017).

## Toward the discovery of key genetic and signaling factors controlling the development of mammalian membrane nanotube networks

Two trivial questions arised from the beginning: what induces the growth of nanotubules and in what direction? We still know much less about the second question, but relatively a lot about the first one. The first reported genetic factor controlling NT growth was *M-Sec* (also known as TNF-induced protein 2, TNFAIP 2) in collaboration with *RalA* small GTPase and the *exocyst complex (*Hase et al. 2009; Kimura et al. 2013*)*. Here, we would like to draw attention to an often neglected point: calling a gene/protein as a key factor in a biological process should be done with caution, due to the high diversity in cell-to-cell gene/protein expression profiles. Further results suggested *LST1* (leukocyte-specific transcript 1) protein to promote assembly of the complex molecular unit inducing nanotubule formation (Schiller et al. 2013a). These two gene products are likely candidates as potential control genes of NT inception, but still need more confirming analysis, referring to a valuable overview on diversity of nanotubes, themselves (Austefjord et al. 2014). Nevertheless, M-Sec has been demonstrated on more and more cell types as a regulator of NT development. Recently, the endoplasmic reticulum-related chaperon *ERp29* has been shown to control TNTs in human osteosarcoma cells. Depletion of this chaperon by silencing dramatically suppressed, while its overexpression increased TNT number, in a rigorously M-Sec dependent manner (Pergu et al. 2019). This suggests that the ER, through the ERp29-M-Sec interaction, likely bridged by other yet unknown proteins, as well, is also indispensable in the regulation of nanotube generation. A detailed structural analysis on M-Sec revealed that its C- and N-terminal parts distinctly contribute to the plasma membrane deformation during the inception of TNTs (Kimura et al. 2016).

It was shown in several murine and human tumor cell types that membrane-bound heat shock protein70 (*mHsp70*), mostly in association with globoyltriaosyl-ceramide (Gb3)-rich membrane domains, contributes to the stabilization of the nanotube networks in these cells (Reindl et al. 2019). Enrichment of mHsp70 in the NTs was found independent of stress, but in tumor cells, their significant redistribution is observable from cytosol toward membrane domains. Neuronal *connexins* (C×36, C×40, C×43, C×45, C×47) were also implicated in the control of mobility and communication of tumor cells via TNTs in an isoform-dependent manner (Rimkutė et al. 2016) (see also Fig. 1A). Reactive oxygen species (*ROS*) were also considered as further inducing factors for cellular protrusions (Liang 2018). Unfortunately, this list seems still far from being complete and thus warrants further investigations to explore more key factors.

## Bacteria and archaebacteria also use nanotubular networks to communicate

It turned out later from experimental works that not only eukaryotes, but prokaryotes can also use such nanotubular connections to communicate with each other or exchange material within a network. Ben-Yehuda and collegues have shown in several works in *B. Subtilis* model that the morphology/structure of bacterial nanotubes is quite different from their mammalian counterparts (Dubey and Ben-Yehuda 2011; Dubey et al. 2016). The altered morphology mostly appears as a consecutive series of fused membrane vesicles vs. a contiguous membrane wall in the case of mammalian nanotubes (see in Fig. 1). Many examples, including other bacterial strains, further confirmed the existence of such communication pathway (Baidya et al. 2018; Bhattacharya et al. 2019; Remis et al. 2014; Wei et al. 2014).

Interestingly, intensive cross-feeding between two independent bacterial strains was also observed via nanotubes connecting these bacterial cells (Pande et al. 2015). This suggests a very early mechanism of bacterial cellular communication in networks. A comprehensive study (Shitut et al. 2019) further confirmed that nanotubular connections may metabolically couple bacterial strains. *Mycoplasma hyorhinis* was shown to induce the formation of nanotubes between NIH3T3 cells by increasing the activity of Rac1 small GTP-binding protein. However, inhibition of Rac-1 function severely reduced *M. hyorhinis* infection, confirming that spreading of infection was basically linked to the development of nanotubular connection network between the cells (Kim et al. 2019). *Schewanella oneidensis MR-1* nanowires were shown as periplasmic extensions of the extracellular electron transport components (Pirbadian et al. 2014). Moreover, novel structures serving for the deployment of outer membrane vesicles were defined as „nanopods” (Shetty et al. 2011). All these observations demonstrate that we are only at the very beginning of exploring and understanding the essence of bacterial networks and their nanotubular communication.

Regarding nanotube networks of bacterial cultures, an important question arised: how the growing nanotubes cross the cell wall when they try to communicate. Baidya et al. (2020) reported a partial answer: the donor cell’s cell wall hydrolase enzymes can facilitate such kind of penetration. In the *Bacillus Subtilis* model, it was shown that these bacteria could communicate with each other using variable routes to establish a communication network, like sensing pheromones, or direct coupling by membrane nanotubes allowing exchange of cytoplasmic content, long-range electrical signals or metabolic coupling (Kalamara et al. 2018).

Some *ancient archaebacteria* (e.g., the hyperthermophilic archaea of the Thermococcus genus) also exhibited nanopod/nanotube-like structures. The *Thermococcus* species produce a high level of extracellular vesicles (EVs) that resemble the composition and properties of their eukaryotic ectosome counterparts (Marguet et al. 2013). *Thermococcus gammatolerans* and *T. kodakaraensis* produce nanotubes containing strings of MVs, very similar to the nanopods of bacteria, whereas *Thermococcus sp. 5–4* produces filaments whose internal membrane is continuous, similarly to most eukaryotic membrane nanotubes.

*Staphylothermus marinus* is a heterotrophic hyperthermophilic archaea that requires elemental sulfur for its growth. Interestingly, these archaebacteria were shown to sequester elemental sulfur from the environment based on a *Right Hand Coiled-Coil Nanotube (RHCC-NT)*, a special protein fragment in the surface layer of the microorganism (McDougall et al. 2019). The cavities in these NTs, adapted to capture small, hydrophobic cyclo-octasulfur can also encapsulate small polyaromatic hydrocarbons (PAHs), which are extremely dangerous to the normal see-life. Since these RHCC-NT structures likely developed under extreme conditions, the authors proposed that they have an extreme chemical stability allowing continuous ability to capture dangerous hydrocarbon derivatives from an aqueous environment, an advantageous property for environment-defense. It was suggested that archaeal nanopods and/or nanotubes could *expand the metabolic sphere around cells and/or promote intercellular communication* between the ancient cells. This might also mean that the intercellular archaebacterial nanotubular bridges could have been general ancestors of the current cellular networks.

## Membrane nanotubes as intercellular highways: Viruses and bacteria both use them to spread

Among the various mammalian cell types exhibiting nanotubular communication, the first described neurons and tumor cells were thoroughly studied including the transport processes (e.g., transport of mitochondria, regulatory micro-RNAs, prions, tau protein, etc.) ongoing via the nanotubes connecting them. In lack of space, here we orient the interested reader to several excellent recent reviews on this subject (Ariazi et al. 2017; Bagheri et al. 2020; Buszczak et al. 2016; Korenkova et al. 2020; Lou et al. 2012a,^ 2018^; Sisakhtnezhad and Khosravi 2015; Thayanithy et al. 2014) and next we will focus on intercellular transport via NTs between cells of the immune system (IS) and its effect on the function of the IS.

It was demonstrated earlier that during infections various virus strains and bacteria can use the “newly discovered” membrane nanotubes, as "tracks”, to spread along with the cells in a certain organ. For example, surfing of *Mycobacterium bovis, bacillus Calmette-Guerin (BCG)4-expressing GFP* was first captured by video microscopy between macrophage cells within a culture (Onfelt et al. 2006). Regarding the viruses, by now many data demonstrated that nanotubular networks can significantly promote the spreading of various virus strains from different families (such as e.g., *HIV*, *influenza A*, *Porcine Reproductive and Respiratory Syndrome Virus (PRRSV), PRV Alphaherpesvirus, *etc*.*) between cells of different mammalian organs (Guo et al. 2014; Jansens et al. 2020; Roberts et al. 2015; Sowinski et al. 2008; Uhl et al. 2019).

The various virus strains use varied protrusion-based strategies for intercellular spreading, including filopodial bridges, microtubule-negative or -positive TNTs, adherent junctions, etc. They can travel as packed virions, or as intact viruses bound to the membrane of the tubules and surf along it, bud from the filopodia membrane and in some cases they can use motor proteins, such as myosin 2A for transportation within the tube, as described in an excellent recent review (Cifuentes-Munoz et al. 2020). Notably, respiratory viruses, such as RSV, human metapneumonia virus (HMPV) or the severe acute respiratory syndrome virus 2 (SARS-CoV-2), also use such filopodial extensions to spread (Cifuentes-Munoz et al. 2020) the induction of which was found associated with increased casein kinase (CK2) signaling. Moreover, budding particles of the SARS-CoV-2 virus together with CK2 were found in filopodia connecting two cells through N-cadherin bridges (Bouhaddou et al. 2020). These interacting filopodia may be further stabilized by N-cadherin/β-catenin clustering (Chang et al. 2018) and transform into a TNT/like structure.

Since several viruses were also implicated in the induction of TNTs or filopodial bridges, a philosophical question arises: whether how long ago the viruses could start to use these “tracks”, and how the “helpful assistance” of these nanotubular highways in their intercellular spreading affected their infectivity at the social level of vertebrates during the evolution. We believe that answers for these questions may lead us to better understand the mechanisms affecting viral infectivity in general at least at the level of cellular communities.

## Intercellular communication via nanotubes may modulate immune functions

Concerning the special immunological aspects of extracellular vesicle or nanotube-mediated intercellular communication, there are a few interesting, "dogma breaker” observations with highly supportive data that urge us to reinterpret some parts of our current basic immunological concepts (György et al. 2011; McCoy-Simandle et al. 2016; Théry et al. 2009; Zaccard et al. 2016). For example, exchange of both MHC-I or MHC-II-bound antigen complexes (Chinnery et al. 2008; Halász et al. 2018; Osteikoetxea-Molnár et al. 2016; Schiller et al. 2013b; Seyed-Razavi et al. 2013) between individual antigen-presenting cells, such as dendritic cells, macrophages or B lymphocytes, may question some strict definitions of the long believed *clonal theory*. This *nanotubular exchange* (a kind of trogocytosis) means that functional, intact, unprocessed MHC/peptide antigen complexes may appear at the surface of individual antigen-presenting cells (APCs) which anyway do not express them momentarily and thus may activate cognate antigen-specific CD4 + or CD8 + T cells. This effect may significantly influence the efficacy of cellular immune response. Such a mechanism can be really significant at some immune-privileged sites where the APCs are spatially located rarely, at a long distance from each other (Chinnery et al. 2008; Seyed-Razavi et al. 2013). On the other hand, such “antigen-spreading” may somehow compensate for the nanotube-mediated microorganism spreading.

In addition, intercellular transfer of B7-1 (CD80), B7-2 (CD86) or programmed death-ligand 1 (PDL-1/CD274) costimulatory/immunoregulatory proteins was also directly detected recently between B cells or macrophages (Halász et al. 2018; Osteikoetxea-Molnár et al. 2016). Such kind of “cross-dressing” of immune cells (Campana et al. 2015; Halász et al. 2018) was proposed as an alternative mechanism to present antigens toward the cognate T cell repertoire and to positively regulate their activity.

Interestingly, earlier it was described in a detailed, thorough assay (He et al. 2007) that T cells and dendritic cells (DCs) may bidirectionally exchange various membrane protein molecules. T cells could acquire for example *Iab, CD11c, CD40 and CD80* molecules from DCs, meanwhile, the DCs acquired *CD4, CD25 (IL-2receptor α chain), CD69 (activation marker)* and even some T cell receptors from the antigen-specific T cells. That time this transfer was highly surprising and hardly interpretable in respect of how this exchange had taken place. Among several others, a similar case was the acquisition of PDL-1 from human APCs to CD8 + T cells (Gary et al. 2012). It was demonstrated that T cells may acquire functional PDL-1 from APCs in an antigen-specific manner, presumably by trogocytosis (mediated mostly by nanotubes or microvesicles) and thus become able to induce apoptosis in PD-1-expressing T cells in their neighborhood. This transfer on the other hand may serve as a momentary regulatory break on the killing branch of the cellular immune response. On the other hand, T cells, upon cross-dressing by APCs (Rainy et al. 2013), may also become antigen-presenting cells, and thus accessible for “self-killing” by other, CD8 + T cells. This process may have an impact on the actual T cell homeostasis and the intensity of T cell response.

Dendritic cells (DCs) (Zaccard et al. 2015) were shown to be exclusively initiated to form nanotubules by the instructive signals of CD40L-expressing T helper cells. It was pointed out that the maturing DCs were uniquely programmed by inflammatory mediators of type-1 immunity to grow NTs. The aforementioned examples all demonstrate that the supercellularity in the immune system provided by, among others, the nanotubular networks may operate in homo- or hetero-cellular interactions alike, and can efficiently regulate/modulate the immune system’s function.

An interesting case has been reported recently for macrophages (Goodman et al. 2019). In a relatively rare but serious lysosomal storage disorder, “cystinosis”, hematopoietic stem and progenitor cell (HSPC)-derived macrophages were shown to develop NT networks and delivered cystinosin-containing lysosomes via these nanotubular channels to the cystinotic cells, resulting in partial reservation of functional tissue integrity. Moreover, transfer of phosphatidylserine (PS)-enriched membrane patches from apoptotic to live macrophage cells (Bittins and Wang 2017) through TNTs was reported, as pro-phagoytic signals.

A recently increasing interest in neutrophil activities in the immune response also appeared in the nanotube research, together with a special, neutrophil-related microbe-killing mechanism, the neutrophil extracellular trap (NET). During the fight of neutrophils against microorganisms, these two mechanisms seem to work hand-in-hand (Galkina et al. 2020, 2013). In this battle, the live and dead/dying neutrophils seem to collaborate enjoying help from neutrophil-derived microvesicles, as well (Timár et al. 2013). The live cells can develop a nanotubular (cytoneme) network, while the dead cells provide "other kinds of weapons”, such as free chromatin and proteins which develop a NET around the cells. Granular bactericide agents may accumulate in the cytonemes, while in the NETs some bactericides may adsorb on the surface of decomposed DNA strains, becoming thus exposed for the infecting microorganism. This complex mechanism can thus eliminate various bacteria by trapping and killing them in a relatively small volume around the neutrophils with high efficiency. Notably, NETs and cytonemes can also be regarded as “double edge sword”, since they may also play some negative roles, as well, for example, in the process of thrombosis and autoimmune diseases (Galkina et al. 2020).

An interesting new aspect of nanotubular communication is the direct intercellular communication of tumor cells with various stages of stem cells or with mature immune cells. For example, CD4 + or CD8 + T cells are known to respond to new tumor antigens and react with cytokine release or antigen-specific cell killing. An earlier study has shown convincingly, using flow cytometry and fluorescent markers, that tumor cells and CD4 + T cells could exchange their own cytosol content, which may result in remarkable functional consequences. While the T cells kept their proliferation capacity, the tumor cell division was largely impeded. These findings were further confirmed in a murine in vivo model (Hardtke-Wolenski et al. 2013). Such hetero-cellular connections suggest that this level of supercellularity may have a serious impact not only on immune functions but also on the communication within the immune-neuronal-endocrine triangle or in their communication with tumor cells. Understanding the details of these pathways, however, warrants much further investigation.

Finally, we would like to point out that sometimes nano-engineering may also help us to understand immunobiological phenomena (Li et al. 2015). Authors observed that bone marrow-derived mast cells intensively started to develop membrane nanotubes upon their costimulation by *engineered IgE-binding antigen* through Fcε-Receptor I and by macrophage inflammatory protein 1α (MIP1 α) through chemokine receptor 1 (CCR1). In the mast cell model rat basophilic leukemia (RBL) cells, lacking CCR1 expression, however, such triggering did not work. This turns the attention to an important point: initiation of nanotube formation is highly orchestrated in most cases and needs well-defined environmental signal(s) to initiate NT growth.

## Biologia Futura: What might be the role of nanotubes historically and in our current biomedicine?

All the aforementioned examples, explored in only two decades of nanotube research, besides shedding light on so far hidden cellular communication pathways, also raised some exciting questions. Carefully analyzing several experimentally demonstrated analogies/similarities between nanotubular signaling communication (e.g., Ca^2+^-, glutamate, etc. signals, electrical coupling) between cells of the nervous (Wang et al. 2012a, 2010) and immune systems (Watkins and Salter 2005), the developing drosophila (Huang et al. 2019; Inaba et al. 2015) and plant cells (Wudick et al. 2018), Nussenzweig concluded that networks of cell membrane nanotube structures can be "ancestors of the nervous system” (Nussenzveig 2019). This hypothesis is in accordance with the above reference works underlying the crucial importance of such communication with their environment for developing cells and also by the uniform employment of nanopods/nanotube-like structures in the communication of prokaryotes (Stempler et al. 2017) and very ancient organism communities, such as archaebacteria (Marguet et al. 2013). Bacterial nanopods together with the extracellular vesicles originating from them might have played a crucial role in intercellular competition of these ancient organisms, in disposal or detoxification of their environment, biomineralization, etc. (Gill et al. 2019). Interestingly, behavior of some bacteria in culture suggested that during the evolution there might have been a real selection "war” between them, because in addition to delivering toxins to each other, the nanotubes also facilitated "looting nutrients" from each other (Dubey et al. 2016; Stempler et al. 2017). Thus, membrane nanotubes were possibly important tools in the selection of prokaryotes.

**Fig. 2:**
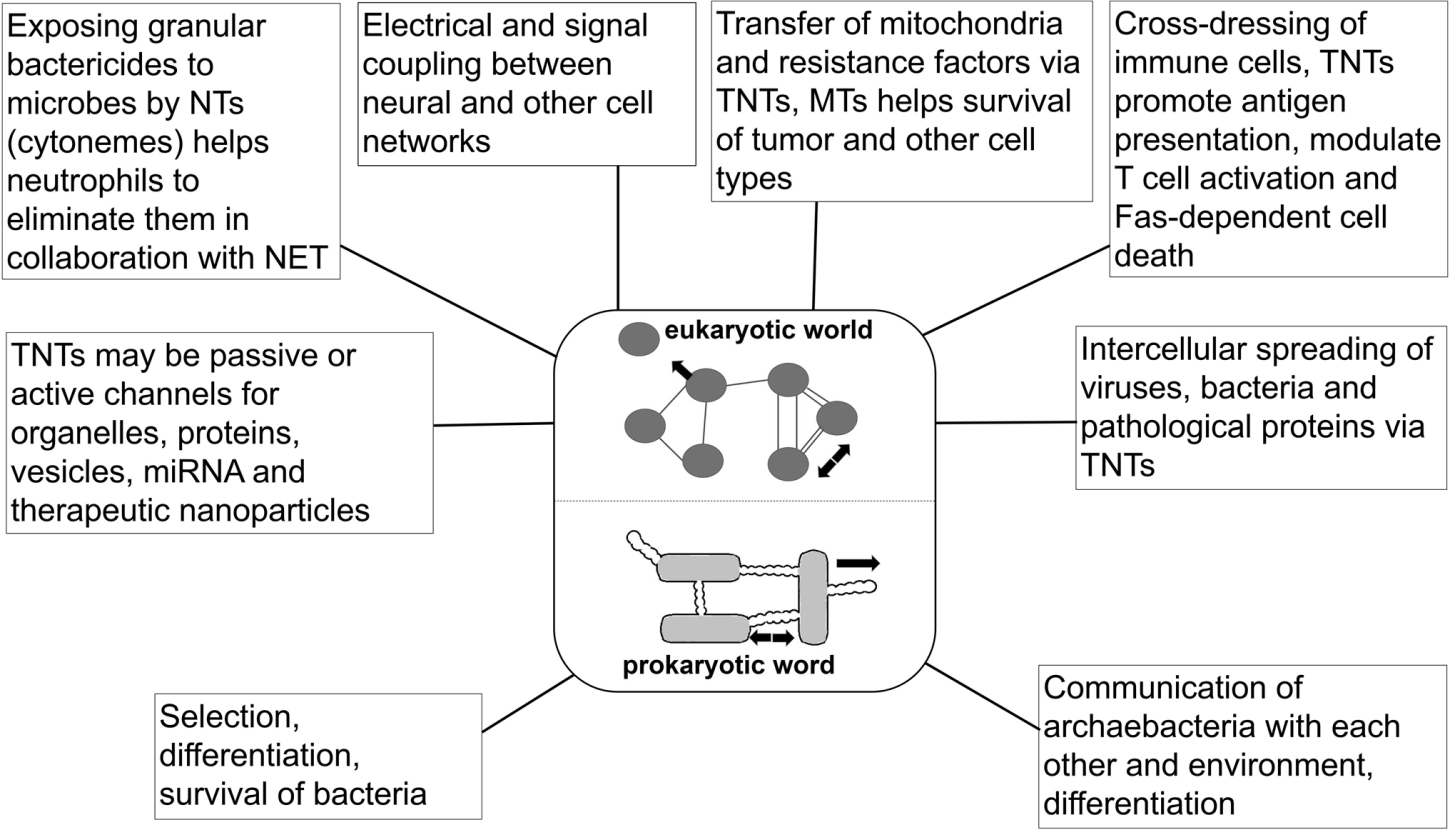
What is the "historical functional role” of membrane nanotubes? Some archae/archaebacteria (more than 3 billion years old species) could also create nanotubular/ nanopod networks between the individual cells suggesting that they communicated this way, resulting in their selection and differentiation. Passing these old times by differentiation, cells of the current eukaryotic world (including the still cohabiting prokaryotic bacteria or the plants) can use nanotubes/nanopods/cytonemes for more complex cellular communication networks (*see boxes around the center of Fig. 2*). These functions include regulatory roles in highly complex organ systems (such as immune system, nervous system, endocrine system and tumors) in the eukaryotic world and also a promising chance for the nanotechnology-based therapeutical approaches to modulate/block certain pathological functions using bio-designed nanoparticles and thereby cure diseases

In nanotubes of *B. Subtilis*, a supposed sensor phosphodiesterase protein, YmdB, was detected which proved to be crucial for the inception of NTs and the intercellular exchange across (Dubey et al. 2016; Stempler et al. 2017). Since this key protein is highly conserved in bacteria and several phyla it would be interesting to check whether they occur and function in other species. An interesting theoretical model (Hooper and Burstein 2014) examining the internalization-based hypothesis of prokaryote–eukaryote transition, based on proton-motive force analysis, suggested, that the intercellular nanopod/nanotube networks could initiate close association of prokaryotes by minimizing the extracellular space, thereby facilitating their evolution toward eukaryotes. Beyond a multifaceted historical role in the development of prokaryotic and eukaryotic cells (Fig. 2), membrane nanotubes are also targets of interest in the current medical therapeutic efforts, especially in tumor- and immune therapies.

It was shown (Ranzinger et al. 2013) that in peritoneal inflammatory processes, occurrence of nanotubes between human peritoneal mesothelial cells (HPMCs) correlates with the marker level (TNFα) of inflammation and showed interdependence with the cytokine action and cellular cholesterol level, as well. Amlodipine, a calcium channel blocker largely reduced the nanotube number between HPMCs. This example raises that carefully designed treatments targeting nanotube inception directly may be useful for clinical therapies, as well.

A latest trend in medical nanotherapy research is the fabrication of various nanometer-sized particles from diverse chemical materials (such as e.g., quantum dots, poly-lactide-co-glycolide (PLG) beads, carbon and silica nanotubes/beads, etc.) and conjugate them with drugs, regulator molecules or antibodies/antibody fragments. Design of such "drug-delivery systems” is continuously expanding (Getts et al. 2014; Rehberg et al. 2016; Sellner et al. 2015; Shi et al. 2010), especially in direction of using biodegradable particles with minimal cytotoxicity and interference with physiological processes. The first high-resolution imaging investigation has shown easy uptake and active, bidirectional intercellular transport of streptavidin-conjugated CdSe/ZnS quantum dots (QDs) with 60 nm diameter on average by cardiac myocytes mediated by membrane nanotubes (TNTs) to distance up to 100 µm (He et al. 2010). Later, an in vivo imaging investigation on mice demonstrated rapid uptake of easily conjugatable carboxyl-derivatized QDs by perivascular and tissue-resident macrophages. The internalized QDs were then detected in long, microtubule-containing nanotubular structures between MHC-II- and F4/80-positive cells (Rehberg et al. 2016), moving bidirectionally along the microtubule tracks presumably with the assistance of kinesin motors. These pioneering studies on intercellular nanoparticle transport pointed to a good chance for nanotube-mediated efficient distribution of drug-delivery nanoparticles in local tissues and its monitoring by whole-body imaging. Further, in vitro investigations demonstrated the existence and movement of silica microparticles in nanotubes connecting endothelial cells (Ferrati et al. 2012), QD-wheat germ agglutinin conjugates in nanotubes between human lung cancer cells (Wang et al. 2012b) or intercellular transport of poly-lactide-co-glycolic acid (PLGA) nanoparticles between neurons (Tosi et al. 2014).

Finally, the immune-modulating approaches based on nanoparticles/ nanovectors are definitely worth mentioning. So far, this approach was mostly tested on macrophages, because they can easily take up these particles by phagocytosis and they meet microbes in the first defense line, thus playing a central role in immune surveillance as well as in resolving the infections. Sellner and collegues proposed a promising nanotherapeutic approach based on the use of *CpG-decorated DNA nanotubes* (Sellner et al. 2015)*.* The design based on the ability of unmethylated CpG sequences of DNA to be recognized by toll-like receptor 9 (TLR9) and thus initiate an innate immune response. This concept was then investigated by microinjection of these “nanovectors” into the skeletal muscle and a subsequent in vivo microscopic monitoring of tissue-resident macrophages in mice. It was found that only the CpG-decorated DNA nanotubes were able to induce leukocyte recruitment and activation of NFκB signaling, but the injection of DNA nanotubes or CpG alone, not. This suggests that such immune-modulating (delivery vehicle) nanoparticles (*IMNPs*) may be potentially good therapeutic agents in the future.

Another promising and multiple-targeted IMP was reported by Getts and collegues (Getts et al. 2014). They found that infusion of negatively charged, immune-modifying microparticles (IMPs) derived from polystyrene, microdiamonds or biodegradable poly(lactic-co-glycolic) acid (PLGA) were easily taken up by inflammatory monocytes through the macrophage receptor with collagenous structures (MARCO). Consequently, these monocytes could not travel anymore to the sites of inflammation to cause serious inflammatory diseases accompanied even with tissue damage, but rather sequestered into the spleen for clearance or dye by apoptosis. Transport of such IMPs across inflammatory monocytes might also be made more efficient by the mediation of membrane nanotubes.

## Conclusion

Membrane nanotubes/nanopods and relative structures might have been employed opportunistically by the evolving multicellular organisms. They served as channels for information/signal and matter exchange between individual cells in larger cellular communities, promoting thereby their differentiation/evolution. In this long-term process, we should give a high credit to an often neglected "lipid evolution”, since without the constantly evolving lipid chemistry (due to the changing environment) and the contiguous membranes this evolution could have run into a dead-end.

Nanotubes also served as platforms of selection processes for both prokaryotes and eukaryotes. Arriving at the current eukaryotic world, they acquired more and more tasks (**see **Fig 2). As a "double edge sword”, they may influence the function of many mammalian organs positively and negatively, alike. The negative influence is mostly due to their potential to promote the intercellular spreading of bacteria and viruses in host cells of different tissues/organs. Interestingly, the positive influence may be coupled to the same property. This is their potential to make the nanomedical treatment strategies more efficient by bridging cellular communities and thereby increasing the efficiency of distribution/spreading of drugs in the targeted tissues.
